# Altercentrism and a change in perspective on the self: The relationships of visuospatial perspective-taking with rumination and mindfulness

**DOI:** 10.1371/journal.pone.0316060

**Published:** 2025-08-20

**Authors:** Massimiliano Conson, Isa Zappullo, Roberta Cecere, Michel Thiebaut de Schotten, Anna Lauro, Vincenzo Paolo Senese, Giusy Piscitelli, Vincenza Cardillo, Luigi Trojano

**Affiliations:** 1 Department of Psychology, University of Campania Luigi Vanvitelli, Caserta, Italy; 2 Groupe d’Imagerie Neurofonctionnelle, Institut des Maladies Neurodégénératives-UMR 5293, CNRS, CEA, University of Bordeaux, Bordeaux, France; 3 Brain Connectivity and Behaviour Laboratory, Sorbonne University, Paris, France; University of Naples Federico II: Universita degli Studi di Napoli Federico II, ITALY

## Abstract

Rumination, a dysfunctional way of thinking, can be counteracted by mindfulness. One leading mechanism through which mindfulness works is a change in perspective on the self, i.e., looking at one’s own mental contents from a decentered perspective. Here, we tested whether a change in perspective on the self is grounded in a well-known cognitive capacity, visuospatial perspective-taking (VSPT), allowing individuals to adopt another’s visuospatial viewpoint. We measured rumination and dispositional mindfulness through self-report questionnaires and evaluated VSPT using a task requiring participants (N = 345) to judge the left/right location of an object with which an agent could interact by gazing, grasping, combining the two cues or adopting a still posture. Cluster analysis identified a group of participants (N = 59) systematically judging the object location from the agent’s (altercentric) perspective. In this group, the main results showed that the altercentric responses in the task condition in which the agent gazed towards the object significantly predicted both mindfulness and rumination. The higher was the proportion of the altercentric responses the higher was the mindfulness score and the lower was the rumination one. These findings provide the first evidence that a change in perspective on the self, involved in dispositional traits like rumination and mindfulness, can be grounded in the altercentric perspective-taking.

## Introduction

Reflecting upon one’s thoughts, feelings and experiences is a critical human ability that can become maladaptive when it takes the form of repetitive negative thinking [1]. A type of maladaptive reflection on the self is rumination; it weakens problem-solving, hampers instrumental behavior [1], and is strongly related to depressive symptoms in several psychopathological conditions [1–3], as well as in nonclinical populations [4–6].

Rumination can be counteracted by mindfulness [7–11]. Indeed, dispositional mindfulness shows an inverse relationship with rumination [12–14]. Likewise, mindfulness training can decrease rumination [15] and reduce depression symptoms [16], thus favouring psychological well-being [9].

The mechanisms through which mindfulness works are still under investigation [7,8,17,18], but one key mechanism is represented by a change in perspective on the self [17,19–24]. Indeed, representing own mental contents from a different, decentered (or a distanced) perspective, can counteract ruminative processing of self-related thoughts decreasing negative affect and favouring psychological well-being [19,25–27]. In their influential review, Hölzel et al. [17] acknowledged that a change in perspective on the self is quite difficult to operationalize and empirical evidence relating to mindfulness is limited. However, interestingly, both cognitive and neural substrates involved in a change in perspective on the self seem to share several commonalities with those implied in the capacity to adopt another person’s visuospatial perspective [17,19,28–30].

Visuospatial perspective-taking (VSPT) is thought to encompass “any process by which one infers something about the visual or spatial properties of a scene in relation to another person or position” [31]. Some hints on the possible similarity between a change in perspective on the self and VSPT might be found in two available studies. One study reported that expert meditators were better than non-meditators on a series of visual attention tasks, among which one requiring to identify ambiguous visual images by considering multiple perspectives [32]. The other study reported that, after a formalized meditation training, the participants showed neurofunctional changes in areas involved in self-related processing (medial orbitofrontal cortex) correlating with performance on a mental imagery task (own-body transformation task) requiring imaging oneself in a spatial position of a schematic human figure [33]. Further hints on a correspondence between a change in perspective on the self and VSPT might be envisaged in models of psychological distancing [26,34], according to which an individual can increase or reduce distance from own mental contents, such as emotional ones, by mentally simulating a new perspective from which representing those contents.

On these bases, we hypothesize that taking another’s, i.e., altercentric, visuospatial perspective could correspond to a change in perspective on the self, a capacity which is highly effective in mindfulness and, vice versa, ineffective in rumination. If this were the case, the higher the capacity to take an altercentric perspective, the easier the possibility to analyse thoughts from a different point of view, with a stronger disposition to mindfulness and a lower disposition to rumination.

To test this hypothesis, we measured disposition to rumination and mindfulness through well-validated self-report questionnaires [35,36] and evaluated altercentric perspective-taking through a behavioral task previously used in studies on VSPT [37–42]. In such a task, participants are required to judge the left/right location of a target object placed in a scene where an agent is present. The participants can spontaneously judge the target location from their own or the agent’s perspective since task instructions do not explicitly require choosing a specific viewpoint. In separate conditions, the agent is shown interacting with the target through different behavioral cues: gazing at the object, grasping it, or both, or neither gazing nor grasping the object.

We can hypothesize that people providing more altercentric responses on the VSPT task can be more able to change perspective on their mental contents, thus being more disposed to mindfulness and less disposed to rumination. Further, since observing an agent interacting with a target object, such as gazing or grasping it, influences altercentric perspective-taking [37–44], by using the above VSPT task, we can test whether the relationship between altercentric responses, rumination and mindfulness is stronger for specific VSPT task conditions.

To pursue this aim, however, we have to reckon with individual differences in performing VSPT tasks, with some individuals being strongly anchored on an egocentric reference frame while others spontaneously adopting an altercentric perspective [45–48]. Available studies demonstrate that VSPT tasks can identify a small number of individuals who tend to systematically provide altercentric responses rather than egocentric ones [38,40,42,45]. Therefore, only after having identified participants providing a considerable and consistent number of altercentric responses (altercentric responders), we could search for possible correlations between the number of such responses and scores on self-report measures of rumination and mindfulness. For this reason, as a first step of the present study, a cluster analysis (Agglomerative Hierarchical Clustering) we used to identify altercentric responders [38]. Then, on data from these participants correlation and regression analyses were conducted to test our hypothesis.

## Methods

### Participants

To ensure adequate statistical power for Agglomerative Hierarchical Clustering (AHC), the minimum sample size should be at least 10*p*K, where p is the total number of variables (predictors) and K the number of expected clusters [49]. Thus, a sample size of 160 participants was the minimum requirement. To estimate the sample size needed to perform the main subsequent statistical analysis, that is correlations and multiple regressions, an a priori power analysis (XLSTAT and g*power) [50,51] was conducted to ensure the detection of an effect of critical interest with a power of 0.80 at an alpha level of 0.05 with an effect size of d = 0.5 (equal to f^2^ = 0.15 for linear multiple regression model) [52]. The analysis indicated that we needed at least 43 participants for the linear multiple regression technique (setting the number of predictors = 4) and 34 participants for the bivariate correlation model (setting r ≠ r0 for H1 and r = 0 for H0) to detect an effect of critical interest. However, as recalled above, for our purposes, these statistical analyses should be performed on participants providing a relevant and consistent number of altercentric responses on the VSPT task. In a previous study on this task [38], a systematic tendency to provide altercentric responses was observed in about 15% of participants only. For this reason, we needed to recruit a sample sufficiently large for identifying a group of altercentric responders of adequate size to perform correlation and regression analyses. So, we recruited 345 right-handed neurotypical participants (191 females and 154 males; mean age = 24.3, SD = 2.8; range: 18–34 years) and we anticipate here that cluster analysis (see Statistical analysis and Results sections) allowed to identify 59 altercentric responders (27 females and 32 males; mean age = 24.8, SD = 2.4; range: 21–31 years).

**Fig 1:**
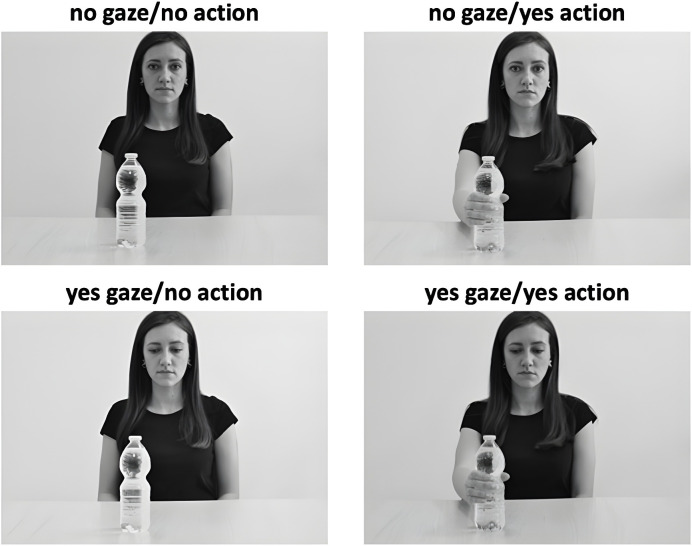
The four scenes used as stimuli of the VSPT task representing the agent (here the female agent is displayed but, during the task, the same scenes were also presented with a male agent) providing different cues of her/his intention to interact with the target object, defining four different conditions: no gaze/no action, no gaze/yes action, yes gaze/no action, yes gaze/yes action condition. Fig 1 provides the images of the actress who posed for the experiment. She is one of the authors of the manuscript and agrees to be in view in the manuscript (see also Participants section).

Individuals could participate if they did not report any past or current history of neurological or psychiatric conditions, neurodevelopmental disorder, or use of substances acting on the central nervous system. The study was approved by the Ethics Committee of the Department of Psychology of the University of Campania ‘Luigi Vanvitelli’ (approval code: N:32/2024) and conducted according to the ethical standards of the Helsinki Declaration. All the participants were blinded to the study’s aims. Written informed consent, previously approved by the Ethics Committee, was obtained from all participants before testing. Furthermore, the individual portrayed in the Fig 1 has given her written informed consent (as outlined in PLOS consent form) to be in view in the manuscript.

### Task and measures

#### VSPT task.

Participants were presented with scenes showing an agent (one female or one male) at a table on which one target object (a bottle) was positioned. Four scenes were devised for each agent (Fig 1). In one scene, the agent had a straight gaze and did not grasp the target (no gaze/no action). In this condition, the agent provided no behavioral cues of her/his intention to interact with the to-be-judged object. In another scene, the agent had a straight gaze but grasped the target (no gaze/yes action). In this condition, the agent provided unclear cues about her/his intention to interact with the target object [39]. In the third scene, the agent gazed towards the target but did not grasp it (yes gaze/no action) and, in the fourth scene, the agent both gazed and grasped the target (yes gaze/yes action). In these two last conditions, the agent provided clear cues of her/his intention to interact with the to-be-judged object [37–40]. Each scene was enclosed in a rectangular 700 x 500-pixel frame. The four scenes for the two agents were presented 12 times in a randomized order (8 trials repeated 12 times for 96 trials).

In each trial, a fixation point (800 ms) was followed by a scene that remained on the screen until participants responded. Task instructions required participants to code target location as left or right, without any mention about the perspective the participants had to assume. The instructions were the following: Where is the bottle? On the left or on the right?”, without further information. Participants responded by pressing one of two buttons on the computer keyboard (“B” for left and “H” for right on the QWERTY keyboard) with their right dominant hand.

Before the task, six practice trials were given and discarded from statistical analysis; participants’ left/right responses and response times were recorded. Since in previous studies using this kind of task, the experimental conditions affected the proportion of participants’ left and right responses rather than response times [37–40], here we considered the proportion of participants’ responses only (see Statistical analysis).

#### Dispositional measures.

**Rumination:** Trait rumination was measured through the self-report Ruminative Response Scale (RRS), a subscale of the Response Style Questionnaire (RSQ) [53]. Treynor et al. [35] revised the original version of the scale [53], due to high confounding with depression symptoms, and developed a version composed of 22 items showing psychometric properties comparable to the original RRS. Each item is rated on 4-point Likert scales ranging from 1 = “almost never” to 4 = “almost always”; the total score is the sum of item scores (range: 22–88) with higher scores meaning higher rates of rumination. In the present study, the Italian version of the 22-item RRS was adopted [54].

**Mindfulness:** The Mindful Attention Awareness Scale (MAAS) [36] was used to assess individual differences in daily mindful states, which are considered the dispositional ability to pay attention to what is occurring in the present moment. The scale mainly deals with mindfulness attention and awareness components rather than other mindfulness-related attributes, such as acceptance, trust, empathy, and gratitude. The scale comprises 15 items, rated on 6-point Likert scales ranging from 1 = “almost always” to 6 = “almost never”; the total score is the mean of item scores (range: 0–6) with higher scores meaning higher disposition to mindfulness. In the present study, the Italian version of the MAAS was adopted [55].

### Procedure

The experimental protocol was administered through an online platform (Google Forms, Google Inc., MountainView, CA, USA). After filling out a personal data form (including sex, age, native language, handedness, and anamnestic data on past and current psychiatric, neurological, and neurodevelopmental conditions), participants were sent two links, one to run the VSPT task through JATOS online platform [56], and another to complete self-report dispositional measures (RRS and MAAS) through the Google Forms online platform. The order of administration of VSPT task and self-report measures was counterbalanced across participants. Data collection started on 27 May 2024 and was completed by 31 October 2024.

### Statistical analysis

#### Preliminary analysis for identifying the altercentric responders.

For each of the four VSPT conditions, we computed the proportion of the altercentric responses to the male and the female agents separately. To identify participants showing a considerable and consistent tendency to provide altercentric responses on VSPT, i.e., the altercentric responders, following Conson et al.’s [38] study, Agglomerative Hierarchical Clustering (AHC) was used. AHC is a multivariate technique for identifying homogeneous subgroups. Here, we used Ward’s method, which minimizes the total within-cluster variance, and the Euclidean Distance [57–59]. Given the specificity of the AHC statistical technique, evaluating the cluster solution’s goodness of fit considers both graphical methods, such as the dendrogram and quantitative parameters [50,58]. The following clustering validity indices were used: i) the Hartigan index, based on the Euclidean within-cluster sum of squares; the maximum difference between clusters is taken as indicating the correct number of clusters in the data; ii) the Silhouette index, the most preferred quality criteria in cluster analysis, based on silhouette values for each observation, measuring how well it fits into the cluster. For the cluster solution, the maximum value of the Silhouette index is used to determine the optimal number of clusters in the data [58,60–63]. Additional validity parameters were also considered: the evolution of the difference between the index of clustering with k clusters and clustering with (k-1) clusters, i.e., H(k-1) – H(k) index; the larger difference indicates the correct number of clusters, and a graphical truncation method of the dendrogram [50]. Before analysis, data across all conditions were normalized as z scores. Then, to gather further confirmation of AHC results, a Discriminant Analysis (DA) was also performed using a stepwise method. DA is a statistical approach that allows modelling a qualitative dependent variable from a new variable (i.e., the discriminant variable), which represents a linear combination of the explanatory variables [64]. This technique uses the discriminate function, based on Bayesian posterior distributions, to provide the posterior probability of membership to the groups for each observation [50,65]. Here, we entered in a stepwise fashion all the VSPT task conditions as independent variables, and the VSPT response group (i.e., clusters emerged from the AHC) as the dependent variable. The Wilks’ Lambda test, with Rao’s approximation, was used to test the hypothesis of equality of the mean vectors for the classes. Additional parameters were also considered: i) The ROC curve (Receiver Operating Characteristics) and its synthetic index, the AUC (Area Under the Curve): AUC values > 0.7 are usually considered as an index of well-discriminating model; ii) the eigenvalues associated with the various factors, and the corresponding discrimination and cumulative variance percentages. Finally, a cross-validation procedure was performed to test the stability of the predictions [50,66,67]. The AHC and DA analysis were performed using XLSTAT package [50].

It is worth anticipating here that most of the participants (non-altercentric responders) provided almost no altercentric response (see Results of Cluster analysis; see also [38]). Furthermore, cluster analysis showed coherent results for the female and the male agents used for each of the four experimental conditions of the VSPT task. This pattern of results allowed to test in the altercentric responders only the relationships of altercentric perspective-taking with rumination and mindfulness.

To test differences between the altercentric responders and the non-altercentric ones on the two dispositional measures, independent samples t-tests were performed. Moreover, to test the relationship between the two dispositional measures within each group, Pearsons’ correlations were also performed.

#### Statistical analyses on the altercentric responders.

Considering results of cluster analysis showing that the agent’s sex did not substantially change the rate of altercentric responses and to get a single index of altercentric responses for each VSPT condition, the mean of the proportion of the altercentric responses for the two actors displayed in the four experimental conditions was computed for each participant and used for the subsequent statistical analyses. Moreover, since Shapiro-Wilk test showed no normal distribution for each condition of the VSPT task (all p < .001) and for the RRS (p = .002), and a trend towards significance for the MAAS (p = .072), the proportion of altercentric responses on the VSPT task was arcsine transformed [68] and the raw scores on the RRS and MAAS were transformed in z scores.

To test the relationships between altercentrism, rumination and mindfulness, first, Pearsons’ correlations of the altercentric responses in four conditions of the VSPT task with MAAS and RRS scores were computed.

To test whether specific VSPT task conditions or combinations between them could represent the best predictors of rumination and mindfulness, two separate multiple stepwise regressions were conducted. This data-driven approach allows for the systematic inclusion or exclusion of variables based on statistical criteria, enhancing model efficiency. Such a method is appropriate when theoretical guidance is limited, or predictors are potentially intercorrelated. In each regression, RRS and MAAS scores were respectively regressed on altercentric responses in the four conditions of the VSPT task.

## Results

### Preliminary analysis for identifying the altercentric responders

**Fig 2:**
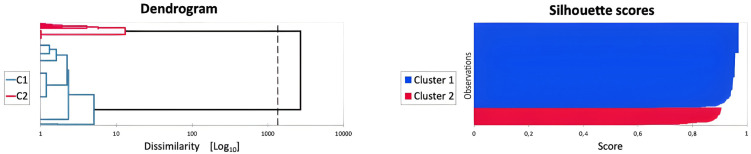
Left column: Dendrogram (the dotted line represents the automatic truncation, leading to two clusters). Right column: Silhouette scores of the two-cluster solution AHC.

Results of the AHC indicated that all indices converged in suggesting a two-cluster solution for the observations (Table 1). Indeed, results showed two main branches in the dendrogram, and the Silhouette scores confirmed that the observations fitted well with the two-group classification (Cluster 1: M Silhouette scores = 0.95, Silhouette range: 0.68–0.97; Cluster 2: M Silhouette scores = 0.87, Silhouette range: 0.59–0.91). Thus, the following two clusters emerged from the data when considering each agent separately: i) Cluster 1: non-altercentric responders (N = 286; Within-cluster variance = 0.09; Average distance to centroid = 0.25; altercentric responders: M = 0.015, SD = 0.022; range = 0–0.22); ii) Cluster 2: altercentric responders (N = 59; Within-cluster variance = 0.65; Average distance to centroid = 0.68; altercentric responders: M = 0.932, SD = 0.070; range = 0.68–1) (Fig 2). Results of DA confirmed that participants were well classified into non-altercentric and altercentric responders based on the predictor variables.

**Table 1:** Evolution of the AHC indices.

Number of clusters	2	3	4	5
*Silhouette index*	0.936	0.850	0.849	0.618
*Hartigan index (H)*	83.89	41.20	40.46	35.75
*H(k-1) – H(k)*	13782.33	42.69	.73	4.70

**Fig 3:**
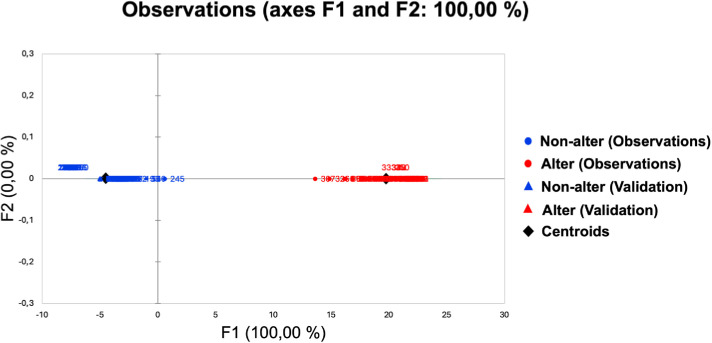
Plot of the observations on the factor axis as a function of the discriminant variable. Non-alter: non-altercentric responders; Alter: altercentric responders.

Results of Wilks’ Lambda test, with Rao’s approximation, showed no equality of the mean vectors for the two groups (Lambda = .011; F(6,288) = 4376.73; p < .001). The discriminant function was significant (Partial R^2^ = .018; F(6,288) = 5.316; Wilks’ Lambda = .011; p < .001) and emerged from 6/8 independent variables (i.e., female: no gaze/no action; female: no gaze/yes action; female: yes gaze/yes action; male: no gaze/no action; male: yes gaze/no action; male: yes gaze/yes action; all p < .001), chosen by the stepwise discriminant procedure. Moreover, results showed that a single factor explained 100% of the variance (eigenvalue: 91.18). As shown in Fig 3, the observations were well discriminated on the factor axis extracted from the original explanatory variables. Cross-validation results showed that 100% of participants were correctly classified, as confirmed by the ROC curve (AUC = 1).

**Fig 4:**
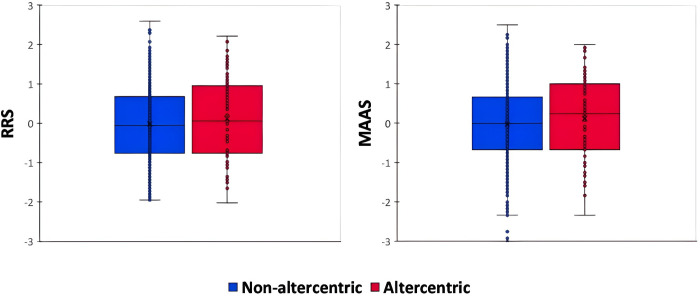
Box plots (boxes represent 25 and 75 percentiles and the solid line inside represents the median) of the scores of the two groups (non-altercentric and altercentric responders) on the two dispositional measures (RRS: left panel; MAAS: right panel).

Results of independent samples t-tests did not show significant differences between the two groups on either of the two dispositional measures (RRS: t(343) = −.98, p = .327; MAAS: t(343) = −.95, p = .341; Fig 4).

**Fig 5:**
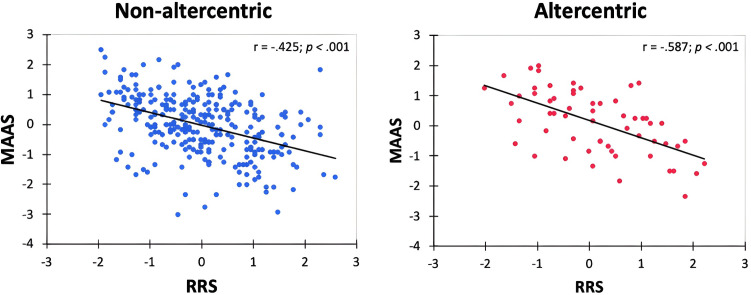
Scatter plots showing significant negative correlations between RRS and MAAS in the non-altercentric (left panel) and the altercentric responders (right panel).

Results of Pearsons’ correlations showed significant negative correlations between RRS and MAAS in both the non-altercentric (r = −.425, p < .001) and the altercentric responders (r = −.587, p < .001) (Fig 5).

### Statistical analyses on the altercentric responders

Descriptive data of the arcsine transformed altercentric responses on the four conditions of the VSPT task (see Supplementary materials in S1 File for results of a two-way repeated-measures analysis of variance showing no significant effects of different intentionality cues on the altercentric responses) and the z scores of the RRS and the MAAS are reported in Table 2 (see Supplementary materials in S1 File for the results of independent samples t-tests showing no within-group differences on RRS and MAAS as a function of altercentric response variability).

**Table 2:** Descriptives (*N* = 59) in the altercentric responders of the arcsine transformed mean altercentric responses in the four conditions of the VSPT task and the z scores of the RRS and the MAAS.

	Mean	SD	Min	Max
**VSPT task conditions**				
** No gaze/no action**	1.26	0.26	0.29	1.57
** Yes gaze/no action**	1.30	0.26	0.67	1.57
** No gaze/yes action**	1.29	0.25	0.72	1.57
** Yes gaze/yes action**	1.32	0.22	0.73	1.57
**RRS**	0.126	1.058	−2.014	2.226
**MAAS**	0.117	1.042	−2.339	1.998

**Fig 6:**
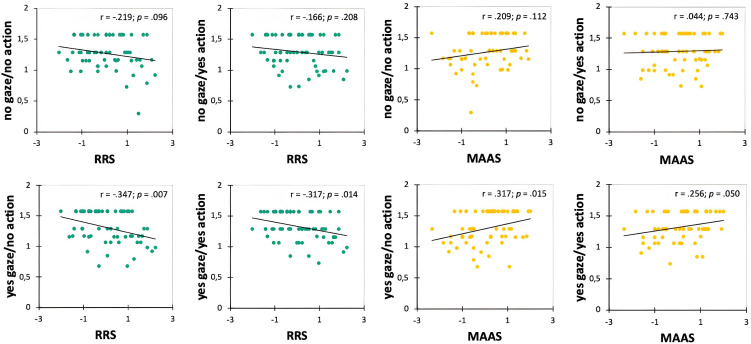
Plots of the Pearsons’ correlations in the altercentric responders between the arcsine-transformed altercentric responses in four conditions of the VSPT task, MAAS and RRS z scores.

Results of Pearsons’ correlations showed significant negative correlations between RRS and the yes gaze/no action (r = −.347, p = .007) and the yes gaze/yes action (r = −.317, p = .014) conditions and significant positive correlations between MAAS and the yes gaze/no action (r = .317, p = .015) and the yes gaze/yes action (r = .256, p = .050) conditions; no other significances were found (Fig 6).

Multiple stepwise linear regression showed that the model for the prediction of RRS score was significant, F(1,57) = 7,812, p = .007, R^2^ = .121, with the yes gaze/no action condition being the only specific predictor included in the final model, indicating that it accounted for a unique and significant proportion of the variance in the dependent variable over and above the other VSPT conditions. In detail, higher altercentric responses on yes gaze/no action condition significantly predicted lower levels of rumination, β = −.347, p = .007.

The model for the prediction of MAAS score was also significant, F(1,57) = 6.31, p = .015, R^2^ = .100, with the yes gaze/no action condition being the only specific predictor included in the final model, indicating that, even for this dispositional measure, altercentric responses to this VSPT condition accounted for a unique and significant proportion of the variance in the dependent variable over and above the other task conditions. Higher altercentric responses on yes gaze/no action condition significantly predicted higher levels of dispositional mindfulness, β = .317, p = .015.

## Discussion

Cluster analysis method used here effectively identified a group of participants providing a considerable and consistent number of altercentric responses on VSPT task. This finding confirms results of a recent study on a large different sample [38] and also fits data from different VSPT tasks [45].

In the altercentric responders we could test the hypothesis that high altercentrism in VSPT was directly related to mindfulness disposition and inversely related to rumination. Correlation analysis showed that altercentric responses in task conditions in which the agent gazed at the target, or both gazed and grasped it, significantly correlated positively with mindfulness and negatively with rumination. Stepwise multiple regression revealed that only the yes gaze/no action condition survived as a specific predictor of both MAAS and RRS scores. Thus, the predictive value of the altercentric responses proved to be stronger for the VSPT condition in which the agent gazed towards the to-be-judged object.

The effect of the agent’s cues on the rate of altercentric responses is largely found in literature and is considered a proof that taking an altercentric visuospatial perspective can occur by relying upon another’s behavioral signals conveying intentionality information [31,37–41,43,44]. Several authors suggested that intentionality cues could favour altercentrism through the activation of embodied processes [37–41,43,44].

Defining embodiment is a complex issue [69–71], but it can be conceived in the context of VSPT as the process by which people can abandon their own position in space and imaging moving towards someone else’s spatial location, thus being able to take her/his viewpoint [37,38,40,41,43,44,69]. In a seminal study using a task similar to the present one, Tversky and Hard [40] demonstrated that intentionality cues, like agent’s gaze and action towards the to-be-judged object, promoted altercentric responses in a comparable way when compared to a condition in which the agent did not provide any intentionality cue (the “mere presence” of the agent). However successive studies did not fully confirmed Tversky and Hard’s [40] results. Therefore, at the present, establishing which is the best combination of agent’s intentionality cues for activating embodiment in VSPT task is an open issue, especially when trying to determine the relative weight of the agent’s gaze and action with respect to conditions in which the agent is merely present in the scenario [37–41,43,44,72,73]. Here, finding that the yes gaze/no action condition was the only specific predictor of both rumination and mindfulness would suggest the relevance of the agent’s gaze in activating the observer’s embodied simulation through which a change in perspective on the self can be achieved. A tentative interpretation of this result could be offered by referring to a model on attention and awareness proposed by Graziano and Kestner [74] according to which the neural structures implied in attending to others’ social signals are also exploited for attending to own mental contents. The authors postulate that one could become aware of another’s mental content by constructing a perceptual representation of the focus of her/his visual attention towards a given target location. The same mechanism is used to become aware of own mental contents by perceiving the target of own focus of attention. Different cues contribute to perception of the focus of someone else’s attention, but the direction of gaze is a pivotal one and a key brain mechanism deciphering this social information is the temporo-parietal junction (TPJ) [75]. Interestingly, neurofunctional data show that the activity of TPJ is implicated in embodying another’s perspective [76–79], as well as in representing others’ and own mental states [80] and attention [81,82]. In a recent study by Christian et al. [81], participants were required to perform two meditation tasks: in the self-focused task, they had to focus attention on their own breath and indicate via a button press when realized their attention had wandered; in the other-focused task, participants watched a video of an actor performing the same task, and they had to press the button if the actor’s attention appeared to have wandered. In both self-focused and other-focused tasks, the authors found a neurofunctional activation in a largely overlapping set of areas, including the TPJ. Accordingly, previous data showed that individuals undergoing formalized mindfulness meditation training displayed a post-training increase of grey matter in TPJ [83]. The activity of TPJ has also been found related to rumination, representing a critical brain site within a connectivity network to be targeted for reducing symptom severity [84,85]. On these bases, we might speculate TPJ be involved in the behavioral relationships we found between altercentric perspective-taking, rumination and mindfulness. In this framework, people providing more altercentric responses might be more capable of embodying another’s focus of attention and exploit this social perception mechanism to look at own mental contents from a different viewpoint, thus being more disposed to mindfulness and less disposed to rumination. Instead, a weak altercentric capacity could be related to the egocentric way of thinking characterising rumination, similarly to what has been suggested for patients with depression [86]. Therefore, a change in perspective on the self, considered as one of the main mechanisms through which mindfulness works [17,19–24], could exploit the same social perception capacity implicated in processing of another’s visuospatial perspective. In this respect, the present results could add to literature on overlaps between self-focused and other-focused processing systems [74,81,82].

Growing data demonstrate that VSPT contributes to cognitive abilities like communication, language, social cognition and self-consciousness [31,47,87,88]. A handful of data also suggest that complex forms of “psychological perspective-taking”, such as to “put oneself in another person’s shoes” [89], are grounded into the basic mechanisms of VSPT [90–93]. For instance, activating embodiment during a VSPT task makes participants to psychologically feel more similar to the person involved in the perspective-taking task and share her/his thoughts [92]. Almost all studies investigating whether complex forms of perspective-taking are grounded into VSPT, including the present one, rely on correlational designs that cannot provide clues on causal mechanisms [90–93] (but see [92]). Empirical demonstration of a shared causal mechanism should derive from experimental methods [92], for instance, testing whether manipulation of the altercentric perspective-taking can affect dispositional measures of rumination and mindfulness. The present correlational results do not speak about the possibility that altercentrism in VSPT is causally involved in the opposite modulation of mindfulness and rumination. However, notwithstanding the correlational nature of the study, here we provided the first evidence that a change in perspective on the self is connected to the capacity of taking an altercentric viewpoint, likely through the activation of embodied processes.

It is important to underline that since the VSPT task used here did not provide participants with explicit instructions about the perspective to be taken, the participants were free of opting for their own or the agent’s perspective during the task. This methodological choice might have induced most participants to believe that the task had to be performed according to the prioritized egocentric reference frame [94,95], whereas only a small proportion of them tended to adopt the agent’s visuospatial perspective systematically. Identifying the psychological traits relating to different egocentric and altercentric preferences [42,45–48] was beyond the scope of the present study. However, it is worth remembering that the altercentric and the non-altercentric responders did not differ in mindfulness and rumination dispositional measures, and that both groups showed a negative correlation between the two dispositional traits, in line with available literature [14,36,96]. Furthermore, we investigated relationships between altercentrism, rumination and mindfulness only in the altercentric responders because in the non-altercentric ones a lack of a sufficient number of altercentric responses prevented us from for testing such relationship. A different VSPT task overcoming this limitation, allowing to capture more fine-grained manifestations of altercentric capacity rather than only identifying consistent altercentric responders, is warranted to generalize the present findings. This could be achieved through a VSPT task allowing to cluster participants not only along a dimension spanning from strong altercentric versus non-altercentric response preference but also along other dimensions, such as the capacity of handling conflicting perspectives [45], or the flexibility in switching between different viewpoints.

Notwithstanding these cautions, the present results offer an important starting point for investigating whether complex forms of perspective-taking, such as decentering or distancing, involved in psychological constructs like rumination and mindfulness, are grounded in systems of mind and brain shared with visuospatial perspective-taking. Implications from this line of research could be of interest in the context of literature on well-consolidated psychological interventions leveraging on decentering [19] to enhance individuals’ capacity to mentally simulate a new perspective from which representing self-related mental contents [17,20,27,97].

## Supporting information

S1 File Supplementary statistical analyses on altercentric responders. (DOCX)

## References

[1] Nolen-Hoeksema S, Wisco BE, Lyubomirsky S. Rethinking rumination. Perspect Psychol Sci. 2008;3(5):400–24. doi: 10.1111/j.1745-6924.2008.00088.x 26158958

[2] Ehring T, Watkins ER. Repetitive negative thinking as a transdiagnostic process. Int J Cogn Ther. 2008;1(3):192–205. doi: 10.1521/ijct.2008.1.3.192

[3] Nolen-Hoeksema S, Watkins ER. A heuristic for developing transdiagnostic models of psychopathology: explaining multifinality and divergent trajectories. Perspect Psychol Sci. 2011;6:589–609. doi: 1745691611419672 26168379 10.1177/1745691611419672

[4] Moberly NJ, Watkins ER. Ruminative self-focus and negative affect: an experience sampling study. J Abnorm Psychol. 2008;117(2):314–23. doi: 10.1037/0021-843x.117.2.314 18489207 PMC2672047

[5] Nolen-Hoeksema S. The role of rumination in depressive disorders and mixed anxiety/depressive symptoms. J Abnorm Psychol. 2000;109(3):504–11. doi: 10.1037/0021-843x.109.3.504 11016119

[6] Nolen-Hoeksema S, Morrow J, Fredrickson BL. Response styles and the duration of episodes of depressed mood. J Abnorm Psychol. 1993;102(1):20–8. doi: 10.1037/0021-843x.102.1.20 8436695

[7] Desrosiers A, Klemanski DH, Nolen-Hoeksema S. Mapping mindfulness facets onto dimensions of anxiety and depression. Behav Ther. 2013;44(3):373–84. doi: 10.1016/j.beth.2013.02.001 23768665 PMC4012250

[8] Desrosiers A, Vine V, Klemanski DH, Nolen-Hoeksema S. Mindfulness and emotion regulation in depression and anxiety: common and distinct mechanisms of action. Depress Anxiety. 2013;30(7):654–61. doi: 10.1002/da.22124 23592556 PMC4012253

[9] Gu J, Strauss C, Bond R, Cavanagh K. How do mindfulness-based cognitive therapy and mindfulness-based stress reduction improve mental health and wellbeing? A systematic review and meta-analysis of mediation studies. Clin Psychol Rev. 2015;37:1–12. doi: 10.1016/j.cpr.2015.01.006 25689576

[10] Kingery JN, Bodenlos JS, Schneider TI, Peltz JS, Sindoni MW. Dispositional mindfulness predicting psychological adjustment among college students: the role of rumination and gender. J Am Coll Health. 2021;71(5):1584–95. doi: 10.1080/07448481.2021.1943411 34437827

[11] Jain S, Shapiro SL, Swanick S, Roesch SC, Mills PJ, Bell I, et al. A randomized controlled trial of mindfulness meditation versus relaxation training: effects on distress, positive states of mind, rumination, and distraction. Ann Behav Med. 2007;33:11–21. doi: 10.1207/s15324796abm3301 17291166

[12] Burg JM, Michalak J. The healthy quality of mindful breathing: associations with rumination and depression. Cogn Ther Res. 2010;35(2):179–85. doi: 10.1007/s10608-010-9343-x

[13] Feldman G, Greeson J, Senville J. Differential effects of mindful breathing, progressive muscle relaxation, and loving-kindness meditation on decentering and negative reactions to repetitive thoughts. Behav Res Ther. 2010;48(10):1002–11. doi: 10.1016/j.brat.2010.06.006 20633873 PMC2932656

[14] Zappullo I, Senese VP, Cecere R, Raimo G, Baiano C, Lauro A, et al. The role of dispositional mindfulness in the impact of repetitive negative thinking on anxiety and depression in people with different autistic-like traits. Mindfulness. 2023;14(4):1005–17. doi: 10.1007/s12671-023-02116-5

[15] Deyo M, Wilson KA, Ong J, Koopman C. Mindfulness and rumination: does mindfulness training lead to reductions in the ruminative thinking associated with depression? Explore. 2009;5(5):265–71. doi: 10.1016/j.explore.2009.06.005 19733812

[16] Shahar B, Britton WB, Sbarra DA, Figueredo AJ, Bootzin RR. Mechanisms of change in mindfulness-based cognitive therapy for depression: preliminary evidence from a randomized controlled trial. Int J Cogn Ther. 2010;3(4):402–18. doi: 10.1521/ijct.2010.3.4.402

[17] Hölzel BK, Lazar SW, Gard T, Schuman-Olivier Z, Vago DR, Ott U. How does mindfulness meditation work? Proposing mechanisms of action from a conceptual and neural perspective. Perspect Psychol Sci. 2011;6(6):537–59. doi: 10.1177/1745691611419671 26168376

[18] Verplanken B, Fisher N. Habitual worrying and benefits of mindfulness. Mindfulness. 2013;5(5):566–73. doi: 10.1007/s12671-013-0211-0

[19] Bernstein A, Hadash Y, Lichtash Y, Tanay G, Shepherd K, Fresco DM. Decentering and related constructs: a critical review and metacognitive processes model. Perspect Psychol Sci. 2015;10(5):599–617. doi: 10.1177/1745691615594577 26385999 PMC5103165

[20] Bishop SR, Lau M, Shapiro S, Carlson L, Anderson ND, Carmody J, et al. Mindfulness: a proposed operational definition. Clin Psychol Sci Pract. 2004;11(3):230–41. doi: 10.1093/clipsy.bph077

[21] Fresco DM, Moore MT, van Dulmen MHM, Segal ZV, Ma SH, Teasdale JD, et al. Initial psychometric properties of the experiences questionnaire: validation of a self-report measure of decentering. Behav Ther. 2007;38(3):234–46. doi: 10.1016/j.beth.2006.08.003 17697849

[22] Kerr CE, Josyula K, Littenberg R. Developing an observing attitude: an analysis of meditation diaries in an MBSR clinical trial. Clin Psychol Psychother. 2011;18(1):80–93. doi: 10.1002/cpp.700 21226129 PMC3032385

[23] Shapiro SL, Carlson LE, Astin JA, Freedman B. Mechanisms of mindfulness. J Clin Psychol. 2005;62(3):373–86. doi: 10.1002/jclp.20237 16385481

[24] Watkins E, Teasdale JD, Williams RM. Decentring and distraction reduce overgeneral autobiographical memory in depression. Psychol Med. 2000;30(4):911–20. doi: 10.1017/s0033291799002263 11037099

[25] Kross E, Ayduk O, Mischel W. When asking “why” does not hurt distinguishing rumination from reflective processing of negative emotions. Psychol Sci. 2005;16(9):709–15. doi: 10.1111/j.1467-9280.2005.01600.x 16137257

[26] Powers JP, LaBar KS. Regulating emotion through distancing: a taxonomy, neurocognitive model, and supporting meta-analysis. Neurosci Biobehav Rev. 2019;96:155–73. doi: 10.1016/j.neubiorev.2018 30502352 PMC6326885

[27] Hayes SC, Pistorello J, Levin ME. Acceptance and commitment therapy as a unified model of behavior change. Counsel Psychol. 2012;40(7):976–1002. doi: 10.1177/0011000012460836

[28] Farb NAS, Segal ZV, Mayberg H, Bean J, McKeon D, Fatima Z, et al. Attending to the present: mindfulness meditation reveals distinct neural modes of self-reference. Soc Cogn Affect Neurosci. 2007;2(4):313–22. doi: 10.1093/scan/nsm030 18985137 PMC2566754

[29] King AP, Fresco DM. A neurobehavioral account for decentering as the salve for the distressed mind. Curr Opin Psychol. 2019;28:285–93. doi: 10.1016/j.copsyc.2019.02.009 31059966 PMC6706318

[30] Lebois LAM, Papies EK, Gopinath K, Cabanban R, Quigley KS, Krishnamurthy V, et al. A shift in perspective: decentering through mindful attention to imagined stressful events. Neuropsychologia. 2015;75:505–24. doi: 10.1016/j.neuropsychologia.2015.05.030 26111487 PMC4631025

[31] Samuel S, Erle TM, Kirsch LP, Surtees A, Apperly I, Bukowski H, et al. Three key questions to move towards a theoretical framework of visuospatial perspective taking. Cognition. 2024;247:105787. doi: 10.1016/j.cognition.2024.105787 38583320

[32] Hodgins HS, Adair KC. Attentional processes and meditation. Consciousness Cogn. 2010;19(4):872–8. doi: 10.1016/j.concog.2010.04.002 20430650

[33] Tomasino B, Campanella F, Fabbro F. Medial orbital gyrus modulation during spatial perspective changes: pre- vs. post-8weeks mindfulness meditation. Conscious Cogn. 2016;40:147–58. doi: 10.1016/j.concog.2016.01.006 26821244

[34] Trope Y, Liberman N. Construal-level theory of psychological distance. Psychol Rev. 2010;117(2):440–63. doi: 10.1037/a0018963 20438233 PMC3152826

[35] Treynor W, Gonzalez R, Nolen-Hoeksema S. Rumination reconsidered: a psychometric analysis. Cognit Ther Res. 2003;27(3):247–59. doi: 10.1023/a:1023910315561

[36] Brown KW, Ryan RM. The benefits of being present: mindfulness and its role in psychological well-being. J Pers Soc Psychol. 2003;84(4):822–48. doi: 10.1037/0022-3514.84.4.822 12703651

[37] Mazzarella E, Hamilton A, Trojano L, Mastromauro B, Conson M. Observation of another’s action but not eye gaze triggers allocentric visual perspective. Q J Exp Psychol. 2012;65(12):2447–60. doi: 10.1080/17470218.2012.697905 22901326

[38] Conson M, Zappullo I, Cordasco G, Trojano L, Raimo G, Cecere R, et al. Altercentrism in perspective-taking: the role of humanisation in embodying the agent’s point of view. Q J Expe Psychol. 2024;78(6):1041–60. doi: 10.1177/17470218241300252 39502001

[39] Furlanetto T, Cavallo A, Manera V, Tversky B, Becchio C. Through your eyes: incongruence of gaze and action increases spontaneous perspective taking. Front Hum Neurosci. 2013;7:445. doi: 10.3389/fnhum.2013.00455 23964228 PMC3740297

[40] Tversky B, Hard BM. Embodied and disembodied cognition: spatial perspective-taking. Cognition. 2009;110(1):124–9. doi: 10.1016/j.cognition.2008.10.008 19056081

[41] Cavallo A, Ansuini C, Capozzi F, Tversky B, Becchio C. When far becomes near. Psychol Sci. 2016;28(1):69–79. doi: 10.1177/0956797616672464 27864372

[42] Ang N, Brucker B, Rosenbaum D, Lachmair M, Dresler T, Ehlis A-C, et al. Exploring the neural basis and modulating factors of implicit altercentric spatial perspective-taking with fNIRS. Sci Rep. 2023;13(1). doi: 10.1038/s41598-023-46205-w PMC10667356 37996437

[43] Brady N, Leonard S, Choisdealbha ÁN. Visual perspective taking and action understanding. Acta Psychologica. 2024;249:104467. doi: 10.1016/j.actpsy.2024.104467 39173344

[44] Lukošiūnaitė I, Kovács ÁM, Sebanz N. The influence of another’s actions and presence on perspective taking. Sci Rep. 2024;14(1). doi: 10.1038/s41598-024-55200-8 PMC10904779 38424102

[45] Bukowski H, Samson D. New Insights into the inter-individual variability in perspective taking. Vision. 2017;1(1):8. doi: 10.3390/vision1010008 31740633 PMC6835961

[46] Bukowski H, Samson D. Automatic imitation is reduced in narcissists but only in egocentric perspective-takers. Acta Psychol. 2021;213:103235. doi: 10.1016/j.actpsy.2020.103235 33321398

[47] Kampis D, Southgate V. Altercentric cognition: how others influence our cognitive processing. Trends Cogn Sci. 2020;24(11):945–59. doi: 10.1016/j.tics.2020.09.003 32981846

[48] Samuel S, Cole GG, Eacott MJ. It’s not you, it’s me: a review of individual differences in visuospatial perspective taking. Perspect Psychol Sci. 2022;18(2):293–308. doi: 10.1177/17456916221094545 35994772 PMC10018059

[49] Qiu W, Joe H. clusterGeneration: Random Cluster Generation (with Specified Degree of Separation), R package version 1.3.8. 2023. Available from: https://cran.r-project.org/web/packages/clusterGeneration/clusterGeneration.pdf

[50] Addinsoft. XLSTAT statistical and data analysis solution. New York, USA. 2022. Available from: https://www.xlstat.com/en

[51] Faul F, Erdfelder E, Lang A-G, Buchner A. G*Power 3: a flexible statistical power analysis program for the social, behavioral, and biomedical sciences. Behav Res Methods. 2007;39(2):175–91. doi: 10.3758/bf03193146 17695343

[52] Lovakov A, Agadullina ER. Empirically derived guidelines for effect size interpretation in social psychology. Eur J Soc Psych. 2021;51(3):485–504. doi: 10.1002/ejsp.2752

[53] Nolen-Hoeksema S, Morrow J. A prospective study of depression and posttraumatic stress symptoms after a natural disaster: The 1989 Loma Prieta earthquake. J Pers Soc Psychol. 1991;61(1):115–21. doi: 10.1037/0022-3514.61.1.115 1890582

[54] Palmieri R, Gapsarre A, Lanciano T. Una misura disposizionale della ruminazione depressiva: la RRS di Nolen-Hoeksema e Morrow. Psychofenia. 2007;10:15–33. doi: 10.1285/i17201632vXn17p15

[55] Rabitti E, Miselli G, Moderato P. Misurare la capacità di restare in contatto con il momento presente: la validazione italiana della “Mindful Attention Awareness Scale”. Psicoterapia Cognitiva e Comportamentale. 2013;3:323–39.

[56] Lange K, Kühn S, Filevich E. "Just Another Tool for Online Studies” (JATOS): an easy solution for setup and management of web servers supporting online studies. PLoS ONE. 2015;10(6):e0130834. doi: 10.1371/journal.pone.0130834 PMC4482716 26114751

[57] Ward JH Jr. Hierarchical grouping to optimize an objective function. J Am Stat Assoc. 1963;58(301):236–44. doi: 10.1080/01621459.1963.10500845

[58] Charrad M, Ghazzali N, Boiteau V, Niknafs A. NbClust package for determining the best number of clusters. R package version 2.0.3. 2014. Available from: http://CRAN.R-project.org/package=NbClust.

[59] Blei D, Lafferty J. Topic models. In: Srivastava A, Sahami M, editors. Text Mining: Classification, Clustering, and Applications. Boca Raton, FL: Taylor & Francis Group; 2009. pp. 71–94.

[60] Hartigan JA. Clustering Algorithms. New York: John Wiley & Sons; 1975.

[61] Rousseeuw PJ. Silhouettes: a graphical aid to the interpretation and validation of cluster analysis. J Comput Appl Math. 1987;20:53–65. doi: 10.1016/0377-0427(87)90125-7

[62] Wang X, Xu Y. An improved index for clustering validation based on Silhouette index and Calinski-Harabasz index. IOP Conf Ser: Mater Sci Eng. 2019;569(5):052024. doi: 10.1088/1757-899x/569/5/052024

[63] Nisha, Kaur PJ. Cluster quality based performance evaluation of hierarchical clustering method. In: 2015 1st International Conference on Next Generation Computing Technologies (NGCT). 2015. pp. 649–53. doi: 10.1109/ngct.2015.7375201

[64] Fisher RA. The use of multiple measurements in taxonomic problems. Ann Eugenics. 1936;7(2):179–88. doi: 10.1111/j.1469-1809.1936.tb02137.x

[65] Ingelfinger JA, Mosteller F, Thibodeau LA, Ware JH. Biostatistics in clinical medicine. New York: Macmillan Publishing Company; 1983.

[66] Jobson JD. Applied multivariate data analysis. Volume II: Categorical and Multivariate Methods. New York: Springer-Verlag; 1992.

[67] Huberty CJ. Applied discriminant analysis. New York: Wiley-Interscience; 1994.

[68] Hogg RV, Craig AT. Introduction to mathematical statistics. 5th ed. Englewood Cliffs, NJ: Prentice Hall; 1995.

[69] Kessler K, Thomson LA. The embodied nature of spatial perspective taking: Embodied transformation versus sensorimotor interference. Cognition. 2010;114(1):72–88. doi: 10.1016/j.cognition.2009.08.015 19782971

[70] Chatterjee A. Disembodying cognition. Lang Cogn. 2010;2(1):79–116. doi: 10.1515/langcog.2010.004 20802833 PMC2927131

[71] Myachykov A, Platenburg WPA, Fischer MH. Non-abstractness as mental simulation in the representation of number. Behav Brain Sci. 2009;32(3–4):343–4. doi: 10.1017/s0140525x09990811

[72] Zhao X, Malle BF. Spontaneous perspective taking toward robots: the unique impact of humanlike appearance. Cognition. 2022;224:105076. doi: 10.1016/j.cognition.2022.105076 35364401

[73] Ward E, Ganis G, McDonough KL, Bach P. Perspective taking as virtual navigation? Perceptual simulation of what others see reflects their location in space but not their gaze. Cognition. 2020;199:104241. doi: 10.1016/j.cognition.2020.104241 32105910

[74] Graziano MSA, Kastner S. Human consciousness and its relationship to social neuroscience: a novel hypothesis. Cogn Neurosci. 2011;2(2):98–113. doi: 10.1080/17588928.2011.565121 22121395 PMC3223025

[75] Guterstam A, Wilterson AI, Wachtell D, Graziano MSA. Other people’s gaze encoded as implied motion in the human brain. Proc Natl Acad Sci USA. 2020;117(23):13162–7. doi: 10.1073/pnas.2003110117 32457153 PMC7293620

[76] Arzy S, Thut G, Mohr C, Michel CM, Blanke O. Neural basis of embodiment: distinct contributions of temporoparietal junction and extrastriate body area. J Neurosci. 2006;26(31):8074–81. doi: 10.1523/jneurosci.0745-06.2006 16885221 PMC6673771

[77] Blanke O, Mohr C, Michel CM, Pascual-Leone A, Brugger P, Seeck M, et al. Linking out-of-body experience and self processing to mental own-body imagery at the temporoparietal junction. J Neurosci. 2005;25(3):550–7. doi: 10.1523/jneurosci.2612-04.2005 15659590 PMC6725328

[78] Martin AK, Kessler K, Cooke S, Huang J, Meinzer M. The right temporoparietal junction is causally associated with embodied perspective-taking. J Neurosci. 2020;40(15):3089–95. doi: 10.1523/jneurosci.2637-19.2020 32132264 PMC7141886

[79] Wang H, Callaghan E, Gooding-Williams G, McAllister C, Kessler K. Rhythm makes the world go round: an MEG-TMS study on the role of right TPJ theta oscillations in embodied perspective taking. Cortex. 2016;75:68–81. doi: 10.1016/j.cortex.2015.11.011 26722994

[80] Saxe R, Kanwisher N. People thinking about thinking peopleThe role of the temporo-parietal junction in “theory of mind”. NeuroImage. 2003;19(4):1835–42. doi: 10.1016/s1053-8119(03)00230-1 12948738

[81] Christian IR, Nastase SA, Yu M, Ziman K, Graziano MSA. Monitoring attention in self and others. J Cogn Neurosci. 2025;:1–11. doi: 10.1162/jocn.a.51 PMC13037715 40392116

[82] Kelly YT, Webb TW, Meier JD, Arcaro MJ, Graziano MSA. Attributing awareness to oneself and to others. Proc Natl Acad Sci USA. 2014;111(13):5012–7. doi: 10.1073/pnas.1401201111 24639542 PMC3977229

[83] Hölzel BK, Carmody J, Vangel M, Congleton C, Yerramsetti SM, Gard T, et al. Mindfulness practice leads to increases in regional brain gray matter density. Psychiatry Res: Neuroimaging. 2011;191(1):36–43. doi: 10.1016/j.pscychresns.2010.08.006 PMC3004979 21071182

[84] Misaki M, Tsuchiyagaito A, Al Zoubi O, Paulus M, Bodurka J. Connectome-wide search for functional connectivity locus associated with pathological rumination as a target for real-time fMRI neurofeedback intervention. NeuroImage: Clin. 2020;26:102244. doi: 10.1016/j.nicl.2020.102244 32193171 PMC7082218

[85] Tsuchiyagaito A, Misaki M, Zoubi OA, Paulus M, Bodurka J. Prevent breaking bad: A proof of concept study of rebalancing the brain’s rumination circuit with real‐time fMRI functional connectivity neurofeedback. Human Brain Mapping. 2020;42(4):922–40. doi: 10.1002/hbm.25268 33169903 PMC7856643

[86] Erle TM, Barth N, Topolinski S. Egocentrism in sub-clinical depression. Cogn Emot. 2018;33(6):1239–48. doi: 10.1080/02699931.2018.1552120 30501568

[87] Frith CD, Frith U. How we predict what other people are going to do. Brain Res. 2006;1079(1):36–46. doi: 10.1016/j.brainres.2005.12.126 16513098

[88] Jääskeläinen IP, Kosonogov V. Perspective taking in the human brain: complementary evidence from neuroimaging studies with media-based naturalistic stimuli and artificial controlled paradigms. Front Hum Neurosci. 2023;17:1051934. doi: 10.3389/fnhum.2023.1051934 36875238 PMC9975546

[89] Epley N, Caruso EM. Perspective taking: Misstepping into others’ shoes. In: Markman KD, Klein WMP, Suhr JA, editors. Handbook of imagination and mental simulation. New York, NY: Psychology Press; 2009. pp. 295–309.

[90] Brunyé TT, Ditman T, Giles GE, Mahoney CR, Kessler K, Taylor HA. Gender and autistic personality traits predict perspective-taking ability in typical adults. Pers Individ Diff. 2012;52(1):84–8. doi: 10.1016/j.paid.2011.09.004

[91] Erle TM, Topolinski S. Spatial and empathic perspective-taking correlate on a dispositional level. Soc Cogn. 2015;33(3):187–210. doi: 10.1521/soco.2015.33.3.187

[92] Erle TM, Topolinski S. The grounded nature of psychological perspective-taking. J Pers Soc Psychol. 2017;112(5):683–95. doi: 10.1037/pspa0000081 28253002

[93] Kessler K, Wang H. Spatial perspective taking is an embodied process, but not for everyone in the same way: differences predicted by sex and social skills score. Spat Cogn Comput. 2012;12(2–3):133–58. doi: 10.1080/13875868.2011.634533

[94] Epley N, Keysar B, Van Boven L, Gilovich T. Perspective taking as egocentric anchoring and adjustment. J Pers Soc Psychol. 2004;87(3):327–39. doi: 10.1037/0022-3514.87.3.327 15382983

[95] Surtees ADR, Apperly IA. Egocentrism and automatic perspective taking in children and adults. Child Dev. 2012;83(2):452–60. doi: 10.1111/j.1467-8624.2011.01730.x 22335247

[96] Raes F, Williams JMG. The relationship between mindfulness and uncontrollability of ruminative thinking. Mindfulness. 2010;1(4):199–203. doi: 10.1007/s12671-010-0021-6

[97] Wells A. Detached mindfulness in cognitive therapy: a metacognitive analysis and ten techniques. J Ration-Emot Cogn-Behav Ther. 2005;23:337–55.

