# Supplementary material for: Altercentrism and a change in perspective on the self: The relationships of visuospatial perspective-taking with rumination and mindfulness
Source: PLoS One. 2025 Aug 20;20(8):e0316060. doi: 10.1371/journal.pone.0316060 (PMC12367188; doi:10.1371/journal.pone.0316060)
Supplement: S1 File — (DOCX) [file pone.0316060.s001.docx]

**Supplementary materials**

*Supplementary statistical analyses on altercentric responders*

First, a two-way repeated measure repeated-measures analysis of variance (ANOVA), with eye gaze (no gaze or yes gaze) and grasping (no action or yes action) as within-subject factors, was conducted on arcsine-transformed responses proportion of altercentric responses to test for the effect of different intentionality cues on the altercentric propensity. Results did not show significant main effects or interaction between the two types of cues (all the p > .05).

Then, independent samples t-tests were performed on the altercentric responders to verify possible within-group differences on the dispositional measures (RRS and MAAS) as a function of altercentric response variability (median split of altercentric responses). Results of independent samples t-tests between the two altercentric subgroups obtained from the median value (μe altercentric responses = .96; Alter1: *N* = 32; M altercentric responses = .89; SD = .07; Alter2: *N* = 27; M altercentric responses = .98; SD = .00) did not show significant differences on either of the two dispositional measures: RRS (t(57) = 1.86, *p* = .068; M Alter1 = .35 ± 1.0; M Alter2 = -.14 ± .97); MAAS (t(57) = -1.70, *p* = .094; M Alter1 = -.09 ± 1.0; M Alter2 = .36 ± 1.04).
